# Evaluating genomic selection and speed breeding for Fusarium head blight resistance in wheat using stochastic simulations

**DOI:** 10.1007/s11032-024-01527-z

**Published:** 2025-01-09

**Authors:** Vinay Kumar Reddy Nannuru, Jon Arne Dieseth, Morten Lillemo, Theodorus H. E. Meuwissen

**Affiliations:** 1 Department of Plant Sciences, Norwegian University of Life Sciences, 1432 Ås, Norway; 2 Graminor AS, 2322 Ridabu, Norway; 3 Department of Animal and Aquacultural Sciences, Norwegian University of Life Sciences, 1432 Ås, Norway

**Keywords:** Wheat, Fusarium Head Blight, Stochastic simulations, Genomic selection, Speed breeding

## Abstract

Genomic selection-based breeding programs offer significant advantages over conventional phenotypic selection, particularly in accelerating genetic gains in plant breeding, as demonstrated by simulations focused on combating Fusarium head blight (FHB) in wheat. FHB resistance, a crucial trait, is challenging to breed for due to its quantitative inheritance and environmental influence, leading to slow progress using conventional breeding methods. Stochastic simulations in our study compared various breeding schemes, incorporating genomic selection (GS) and combining it with speed breeding, against conventional phenotypic selection. Two datasets were simulated, reflecting real-life genotypic data (MASBASIS) and a simulated wheat breeding program (EXAMPLE). Initially a 20-year burn-in phase using a conventional phenotypic selection method followed by a 20-year advancement phase with three GS-based breeding programs (GSF2F8, GSF8, and SpeedBreeding + GS) were evaluated alongside over a conventional phenotypic selection method. Results consistently showed significant increases in genetic gain with GS-based programs compared to phenotypic selection, irrespective of the selection strategies employed. Among the GS schemes, SpeedBreeding + GS consistently outperformed others, generating the highest genetic gains. This combination effectively minimized generation intervals within the breeding cycle, enhancing efficiency. This study underscores the advantages of genomic selection in accelerating breeding gains for wheat, particularly in combating FHB. By leveraging genomic information and innovative techniques like speed breeding, breeders can efficiently select for desired traits, significantly reducing testing time and costs associated with conventional phenotypic methods.

**Supplementary Information:**

The online version contains supplementary material available at 10.1007/s11032-024-01527-z.

## Introduction

Wheat (*Triticum aestivum* L.) is the most cultivated cereal crop in Europe, and among the top three cereals globally. There is an increasing demand of food for a rapid growing population, there will be demand of about 50% more food required by the year 2050 (FAO 2017). This demand can be achieved through sustainable ways by combing integrated management of pests and diseases, adaptation to climate changes and frequent abiotic stresses. For this, breeders are continuously developing more efficient germplasm improving both yield, quality, and disease resistance. Savary et al. (2019) estimated that roughly 21.5% of the present-day crop losses are due to biotic stresses such as pests and disease. This risk of crop losses from plant diseases and pests should be reduced together with a reduction of the use of pesticides and fungicides. Hence, new cultivars are needed with reduced environmental impact that combine excellent disease resistance with productivity and end-use quality.

Both climate change and increasing food demand give rise to challenges in wheat breeding. New cultivars must be developed quickly to accommodate new conditions, both ecologically and economically. For decades, conventional breeding processes based on phenotypic selection have been practiced where the best performing lines were selected based on their performance in field trials regarding yield, disease resistance, and agronomic traits. It is time and resource-consuming, typically it takes approximately 10 to 12 years to develop a new cultivar. Genetic gains are low with phenotypic selection due to the long generation interval and low heritability of traits observed on single plants or small unreplicated field trial plots in early segregating generations of the breeding cycle. The field testing is also labour intensive and costly.

To accelerate breeding and reduce costs, novel approaches are being developed. The availability of high-density low-cost marker genotyping platforms makes genomic prediction, and selection feasible. Therefore, genomic selection, first suggested in 2001 (Meuwissen et al. 2001) can be implemented for the prediction of breeding values of progeny lines without costly phenotyping, saving time and money, increasing intensity of selection as well as accuracy of trait prediction. Genomic prediction aims to utilize genetic information, such as SNP markers to enable prediction of the breeding values of new breeding lines based on their genotypes. Coupled with state-of-the-art data analysis, including machine learning and multi-trait prediction models, genomic selection has proven to be a potent tool for breeding, decreasing the necessity of manual evaluation at great costs. Genomic selection methodology works well for the traits that are high in heritability, this made breeding industries to invest in this technology (Crossa et al. 2017). Genomic selection is now being applied in many animal and plant breeding programs across the globe. It is being widely used in plant breeding programs for many crops, such as maize, wheat, rice, and vegetables (Beyene et al. 2015; Duangjit et al. 2016; Guo et al. 2020; Rutkoski et al. 2014a, b; Song et al. 2017; Spindel and Iwata 2018; Xu et al. 2018). Genomic selection has so far been effectively tested for improving yield (Michel et al. 2017; Norman et al. 2017; Poland et al. 2012a, b; Song et al. 2017), stem rust (Rutkoski et al. 2014a, b) and Fusarium head blight resistance (Arruda et al. 2016; Jiang et al. 2017) in wheat. Despite being successfully demonstrated in various breeding programs, still many breeders tend to follow conventional methods of phenotypic selection. High initial costs such as genotyping costs, training required, and other factors related to prediction accuracies are restricting the breeders to use genomic selection. Especially for traits which are low in heritability and affected by G x E interaction, the prediction accuracies can be low (Fu et al. 2017). Much research is conducted to overcome this drawback of low prediction accuracies (Guo et al. 2020; Lopez-Cruz et al. 2015; Sandhu et al. 2021).

Speed breeding (Ghosh et al. 2018; Watson et al. 2018) is a quite recent rapid generation advancement method, which is based on growing plants at high temperatures in combination with high light intensities and long photoperiods to shorten the generation intervals. Speed breeding has been tested in many crops, and in spring wheat six generations can be achieved in a single year. Genomic selection can be integrated with speed breeding which can further help to reduce the length of the breeding cycle. This idea was investigated by Kai Peter Voss-Fels et al. (2019a, b) using computer simulations.

Stochastic simulations which are used to evaluate breeding programs virtually are gaining increasingly attention in plant breeding. These simulations can be used to develop and simulate entire breeding programs artificially that mimic real breeding programs with user-defined parameters. Such simulations can help breeders in predicting the outcomes of alternative breeding schemes. Integrating genomic selection in plant breeding programs and testing their potential can be assessed with the help of these simulations prior to its implementation in real breeding programs as this implementation involves big investments in terms of labour and time, and the probability of satisfactory results should be maximised.

However, integrating genomic selection in plant breeding programs has the potential of increasing genetic gains per unit time, i.e. more genetic gain with reduced length of breeding cycle (Crossa et al. 2017). There are several factors involved in implementing genomic selection in breeding programs such as experimental design, population size (plot size, number of replicates, founder population size), and trait genetic parameters (heritability, Genotype x Environment interactions, different statistical models). To take all these factors into account, the number of alternative breeding strategies can become very large. Different breeding strategies and their alternatives may be tested with the help of stochastic computer simulations. As mentioned earlier, implementing alternative genomic selection breeding schemes, and comparing them in normal field conditions can be laborious and time-consuming. Simulation studies will model these alternative breeding strategies and compare their potentials against each other. Also, they help in providing in-depth understanding of factors that have specific impacts on each of the breeding schemes and the factors contributing to increased genetic gains. By this means it helps breeders to select breeding strategies in terms of increased selection accuracies and genetic variance. In simulation studies, any number of factors that contribute to the result can be defined as per user specifications and one can observe which of those factors contributes to the desired predictions. There are a number of tools available in the field of plant and animal breeding where both animal and plant breeding programs can be simulated. Some of the ready-to-use available softwares for performing stochastic simulations in breeding programs include QuMARS, AlphaSimR ADAM-plant, and BSL (Ali et al. 2020; Gaynor et al. 2021; Liu et al. 2019; Yabe et al. 2017). There is no accurate deterministic method to compare which simulation software is better over others in performing simulations and each one has their own advantages and disadvantages. Most of them have user-defined features, where the user can simulate an entire population of individual plants or animals, and this artificial breeding program can be defined as per user’s desire.

We used the selection index approach in this study because the breeders generally evaluate several traits of interest and the correlations between such traits help to maximize the breeders aim of selection. It is highly relevant for FHB resistance in wheat, where visual FHB severity and mycotoxin content is negatively correlated with both plant height (PH) and anther extrusion (AE). Skinnes et al. (2010) detected a consistent and negative correlation between AE and FHB disease severity, and AE and deoxynivalenol (DON) content in the Arina x NK93604 mapping population. A study by Lu et al. (2013) performed on the mapping population Shanghai3/Catbird x Naxos confirmed the negative correlation between AE and FHB, and the QTL analysis further confirmed the relationship; eight out of ten AE QTL detected in the study coincided with FHB severity. Kubo et al. (2013) demonstrated that partially extruded anthers were a good source for FHB infection, while rapid extrusion and ejection of the anthers contributed to the avoidance of infection by FHB. A meta-analysis performed by Mao et al. (2010) confirmed a negative association between PH and FHB, where coincident QTL for PH and FHB were detected on chromosomes 2D, 3A, 4B, 4D and 7A. QTL for both FHB disease severity and DON content can serve as valuable sources for disease resistance breeding in wheat. SNP markers closely linked to the resistance QTL could be further tested and used in resistance-breeding for FHB and DON resistance.

This study aimed to examine the potential of genomic selection to increase genetic gains over conventional phenotypic selection by using stochastic simulations based on real breeding program data and a simulated dataset. We also compared the effects of integrating speed breeding with genomic selection relative to genomic selection alone. To our knowledge, this is the first study to compare breeding schemes for resistance to a complex disease like FHB in wheat using stochastic simulations.

## Materials and methods

The required datasets for the present study were generated using a recent and well-established R package “AlphaSimR” (Gaynor et al. 2021) in R version 4.0.3 (R Core Team 2021), which is suitable for stochastic simulation of plant and animal breeding schemes.

In our study, we used two different datasets, one of them is based on real-life breeding program data, while the other was a simulated example imitating the wheat breeding program. The real-life data was based on the NMBU spring wheat panel developed at the Norwegian University of Life Sciences (NMBU) in collaboration with the Norwegian breeding company Graminor and used for genome-wide association studies (GWAS) of FHB resistance (Nannuru et al. 2022). Hereafter, we refer to the dataset from the real-life breeding programs as MASBASIS dataset and the simulated example on wheat breeding program as EXAMPLE dataset. Four different types of breeding schemes were simulated – three of them are based on implementing genomic selection and the other one is a regular conventional phenotypic selection scheme. All four breeding schemes were evaluated for their breeding performance. For doing this, an initial burn-in phase was used, which served as a starting point for comparison of the breeding schemes during the breeding advancement phase. The burn-in phase was simulated for 20 years, and the breeding advancement phase was simulated for the following 20 years. We compared the three genomic selection-based breeding schemes with phenotypic selection to evaluate the potential benefits of implementing genomic selection in plant breeding schemes. Two different selection strategies were used in each breeding scheme, i.e. selection based on a single trait and using a selection index of multiple correlated traits. A detailed description of material and methods used in this simulation study is provided below.

### Simulation of founder populations, genetic values, and phenotypes

#### Genome and founder population

The genome sequence was simulated using real genotypic data from a set of wheat breeding lines called MASBASIS developed for more than a decade at NMBU and Graminor (Nannuru et al. 2022). The genome consisted of 21 pairs of chromosomes and they are ordered based on genetic lengths from the genetic map in cM (Poland et al. 2012a, b). The genome for the EXAMPLE dataset was simulated using the Markovian coalescent simulator MaCS (Chen et al. 2009), consisting of 21 chromosomes. Each chromosome has a length of 1.43 Morgans and a physical length of 8 × 10^8^ base pairs. The values for recombination rate used were 1.43 Morgans/8 ´x 10^8^ base pairs = 1.8 × 10^–9^ per base pair, mutation rate was 2 × 10^–9^ per base pair and an effective population size of 50 that increases up to 32,000 at 100,000 generations back in time. These values were chosen following the evolution pattern in wheat as described and used in other studies (Gaynor et al. 2017; Peng et al. 2011; Thuillet et al. 2005). The number of single nucleotide polymorphisms (SNPs) per each chromosome in the MASBASIS dataset are as follows: 1231 (1A), 1295 (2A) 1168 (3A), 765 (4A), 1425 (5A), 1202 (6A), 1530 (7A), 1268 (1B), 1465 (2B), 1451 (3B), 645 (4B) 1468 (5B), 1155 (6B), 1042 (7B), 452 (1D), 500 (2D), 333 (3D), 139 (4D), 360 (5D), 355 (6D), 362 (7D). The founder population for both datasets were produced using the generated genome sequences and it served as parents for crossing and generated further generations. The procedure followed for the simulation of founder populations is based on the methodology described by Faux et al. (2016) and Gaynor et al. (2021) in their respective simulation softwares called AlphaSim and AlphaSimR. The number of bi-allelic quantitative trait nucleotides (QTNs) or markers assigned from the genetic architecture of traits are equal in both datasets. A sample of 100 QTNs and 200 SNPs were randomly assigned to each chromosome to simulate the genetic effects of the traits, which made a total of 2100 QTNs or 4200 SNPs.

#### Genetic values and phenotypes

FHB disease related traits and other agronomic traits were simulated: FHB disease severity and deoxynivalenol (DON) content, and anther extrusion (AE), plant height (PH) and Days to heading (DH). Both FHB and DON were adjusted for PH and DH by using PH and DH as covariates in multiple regression model. Genetic values of each of these traits were calculated adding up the genetic effects of the particular trait across the loci affecting the disease resistance of FHB disease. A standard normal distribution was used to sample the genetic effects for each trait, and which sums to a total genetic variance of 1 (${\upsigma }_{g}^{2}$ = 1) and with a mean of 0. The genetic values were simulated by adding up the allele effects for each of the traits across the assigned QTNs, individually in both datasets.

Phenotypic values for each of these three traits were generated by adding random error to genetic values. The random error was defined by the heritability level at each stage of the breeding cycle. Random error were sampled from the normal distribution with a mean value of zero and an error variance. Heritablities for each trait used in this study were based on realistic heritabilities obtained from the MASBASIS dataset: DON content (0.69), FHB severity (0.50) and AE (0.74). Trait correlations were used in simulating the phenotypes (Table 1). Both the heritabilities and trait correlations were taken from the GWAS study on MASBASIS germplasm for the traits AE, DH, DON content, FHB disease severity and PH. MASBASIS material was tested for five years and in four different field locations, where FHB disease scoring was conducted when the stem started to turn yellow by visually assessing the percentage of infected spikelets relative to total number of spikelets and DON content was analyzed for each harvested plot sample evaluated using Gas Chromatography – Mass Spectroscopy method. DH was scored when 50 percent of heads had emerged, and PH was measured in centimeter. AE was scored visually by a scale 0 – 9, where 0 means no AE and 9 means full AE. Not all the traits were evaluated in all the field trails or locations (Nannuru et al. 2022). These phenotypes were simulated based on the details described in the AlphaSimR package (Gaynor et al. 2021).

**Table 1:** Pearson’s correlation coefficients between anther extrusion (AE) days to heading (DH), deoxynivalenol (DON) content, fusarium head blight (FHB) severity and plant height (PH) of the MASBASIS dataset (Nannuru et al. 2022)

	AE	DH	FHB	PH
DH	0.054	–		
FHB	−0.48***	−0.013	–	
PH	0.16**	0.0013	−0.43***	–
DON	−0.47***	0.38***	0.66***	−0.31***

***P < 0.0001, **P < 0.001, *P < 0.05

### Simulation of breeding programs and the genomic selection model

#### Phenotypic selection

**Fig. 1:**
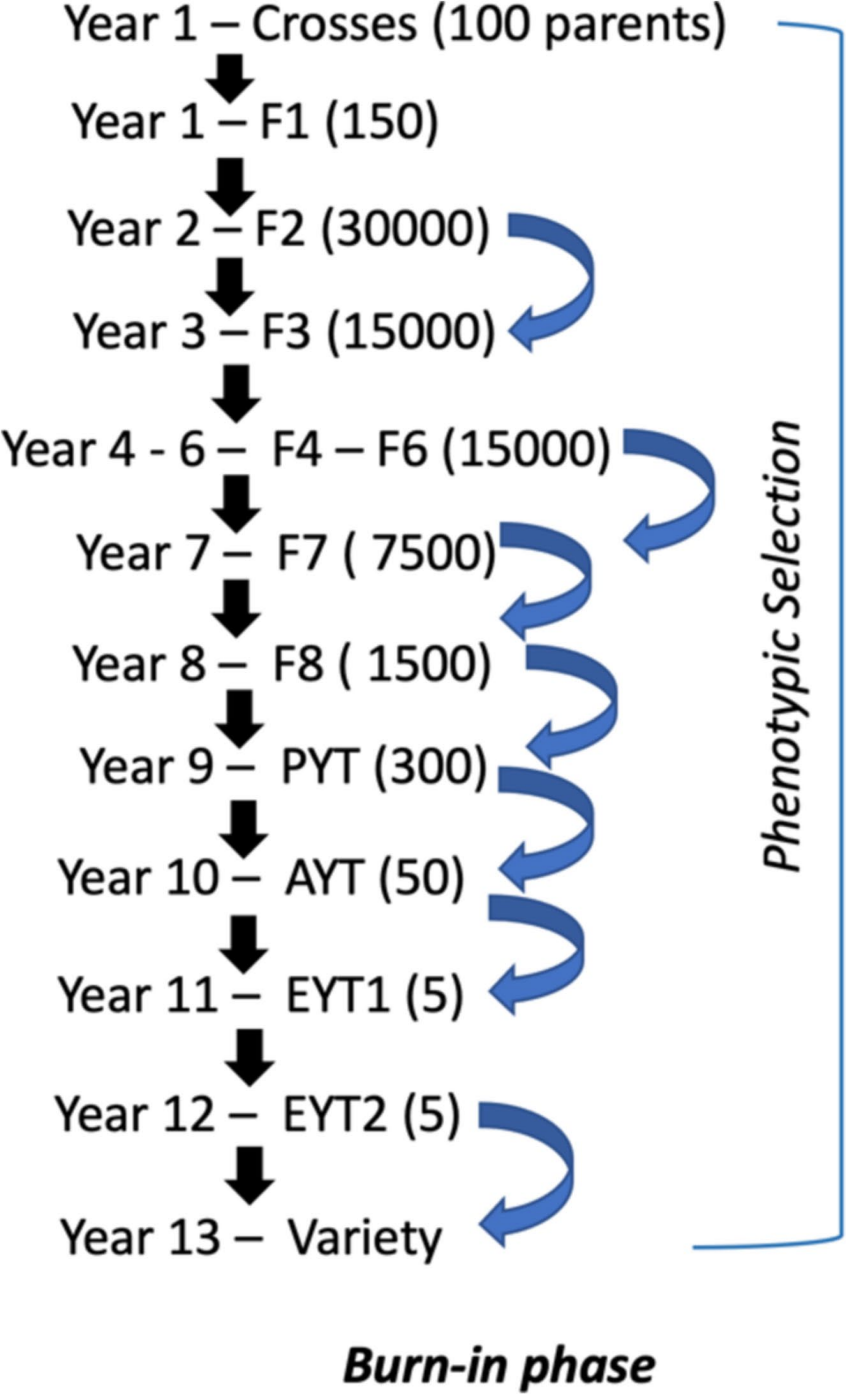
Graphical representation of the simulated phenotypic selection, which was used in the Burn-in phase, and the phenotypic selection breeding scheme. The figure depicts number of generations within a cycle including the years. Selection is done by conventional phenotypic method

In phenotypic selection, each breeding cycle included crossing of founder population (crossing block parents) followed by the selection based on the phenotypic performance of the lines in the field in specific generations after attaining homozygosity (Fig. 1). The conventional selection or the common pipeline used for different breeding programs used in the current study were based on realistic numbers of crosses, population sizes and generation intervals as commonly used in small to medium-sized spring wheat breeding programs in wheat, i.e. each year one new breeding cycle was started by crosses being made during the winter, and the following generations being planted and evaluated in the field with one generation per year in the conventional phenotypic selection scheme (the number of years per each cycle of different alternative breeding programs depends on using genomic selection and speed breeding). In each year, parents in the crossing block were updated by the best performing selected candidates from the advanced yield trial stage (Fig. 3). Selection of candidates is done by two different selection strategies used in this study, that will be described later in this section. This selection is based on phenotypic values involving phenotypic selection, otherwise estimated breeding values (EBVs) are used in genomic selection related breeding program schemes.

##### Crossing

In the first year, a founder population of 100 parental lines were used to make the crosses in the crossing block to produce 150 crosses. These 150 crosses were chosen randomly from 4900 pairwise crossing combinations.

##### Generation F1

Following the crossing, the progeny plants from each cross were grown in head rows in the same year.

##### Generation F2

In the second year, two hundred F2 seeds from each F1 plot were space planted. A visual selection for some agronomic traits such as plant height (moderately tall plants) and heading date, was assumed as is done in real breeding schemes. 15000 candidate plants were selected. Here and in later generations, selection was based on a single trait or a selection index approach described later in this section and then advanced to the next generation.

##### Generation F3 – F6

The selection candidates from F2 generation are advanced using the single seed descent method from year three through the sixth (F3 – F6). The single seed descent (SSD) method was used as it is a cost-efficient method to attain homozygosity. The number of plants remain the same throughout these generations, 15,000 individual plants each generation. Each generation, one seed is advanced from the previous generation plant and evaluated in the field by space planting. Whereas, in the F6 generation, 7500 individuals are selected and advanced to the next generation.

##### Generation F7

In F7, the selected 7500 lines are advanced and evaluated for FHB disease traits in the field using small plots. Based on best performance for the trait of importance, 1500 lines are selected and advanced to the next generation.

##### Generation F8

In the eighth year, the selected 1500 lines are advanced and evaluated for FHB disease traits in the field using small plots. Based on best performance for the trait of importance, 300 lines are selected and advanced to the next generation.

##### Preliminary Yield Trial (PYT)

The 300 lines from the previous generation are evaluated in three locations in year eight. The 50 best performing lines are advanced and evaluated for FHB disease traits in the next field trial. The 20 best performing candidates from the selected lines are used for next year’s crossings to replace old parents with the least performance in the crossing block.

##### Advanced Yield Trial (AYT)

The 50 lines from the previous generation are evaluated in five locations in year nine. Best performing 5 lines are advanced and evaluated for described FHB disease traits in the next field trial.

##### Elite Yield Trial (EYT) 1

In the eleventh year, the selected 5 lines from the previous generation are evaluated in ten different locations and advanced for another year of testing.

##### Elite Yield Trial (EYT) 2 and Variety release

The 5 lines from EYT1 are re-evaluated in the year twelve, to avoid any error and to choose the best one from the evaluation of two years. The selected line is submitted to official variety trials for release in the last year of the breeding cycle.

#### Genomic selection-based breeding schemes

**Fig. 2:**
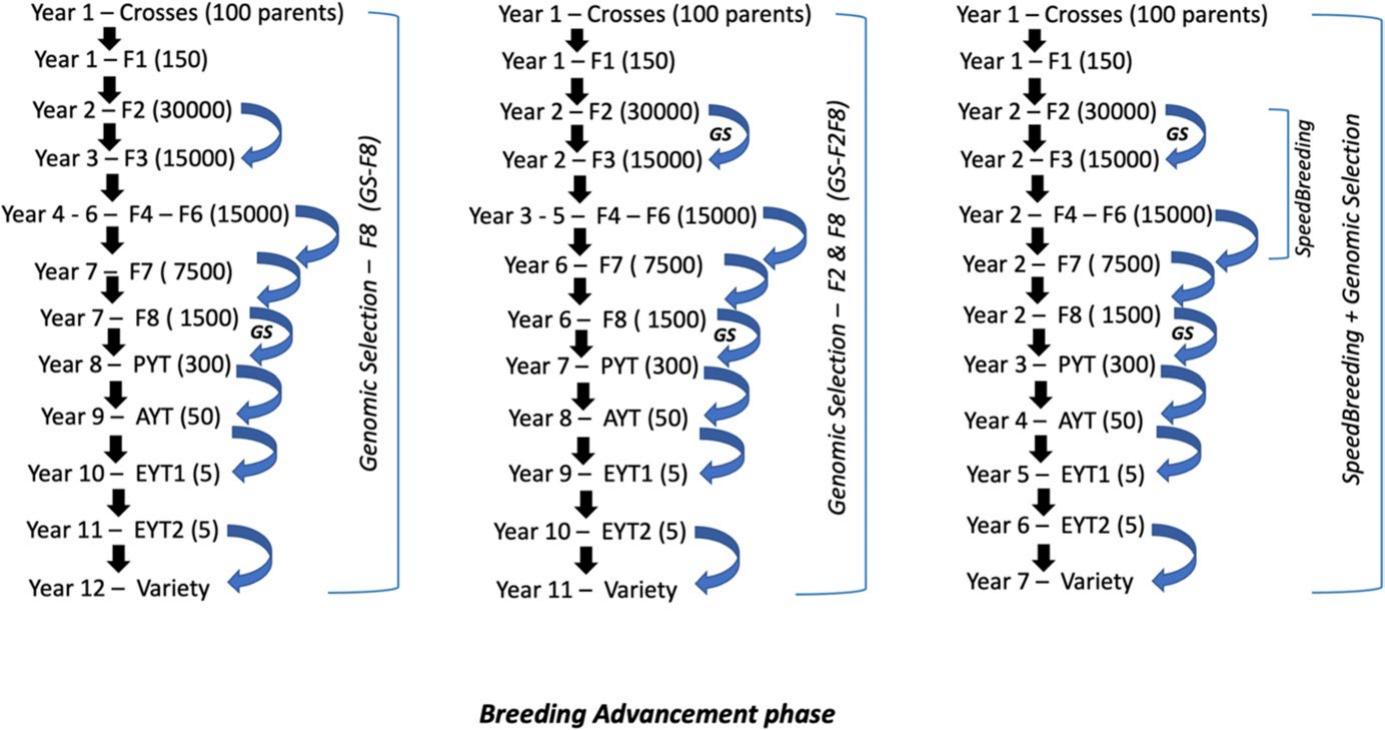
Graphical representation of the three alternative genomic selection breeding schemes used in the breeding advancement phase: Genomic selection F8, Genomic selection F2 & F8, and SpeedBreeding + GS. Each breeding scheme depicts the number of generations within a cycle including the years. GS translates to genomic selection

Three alternative breeding schemes were designed (Fig. 2a, b and c) and simulated, which implemented genomic selection with the use of genomic estimated breeding values (GEBVs). They included GSF8, GSF2F8 and SpeedBreeding + GS, which is an integration of speed breeding and genomic selection. These schemes are described below in detail including the genomic selection model used in this study.

##### GSF8

In the GSF8 breeding scheme, selection of lines at generation F8 was based on GEBVs. 20 of the best performing lines from selected candidates of PYT generation will be added to the crossing block and replace old parents for crossing in the next year. This breeding scheme implements one round of genomic selection with generation advancement in the greenhouse during winter, which reduces the breeding cycle by one year compared to the conventional breeding scheme.

##### GSF2F8

In the GSF2F8 breeding scheme, selection of lines at generation F2 and F8 was based on GEBVs. 20 of the best performing lines from selected candidates of PYT generation will be added to the crossing block and replace old parents for crossing in the next year. This breeding scheme implements two rounds of genomic selection with generation advancement in the greenhouse during winter, which reduces the breeding cycle by two years compared to the conventional breeding scheme.

##### SpeedBreeding + GS

The *SpeedBreeding* + *GS* breeding scheme is like the GSF2F8 breeding scheme in which genomic selection is performed in generation F2 and F8, but speed breeding is used to advance the generations from F2 to F8, shortening the breeding cycle by another two years compared to the GSF2F8 scheme and a total of four years compared to the conventional phenotypic selection scheme.

#### Genomic selection model

The initial training population during the start of advancement phase consisted of genotypes of PYT stage from the last five years of the burn-in period, a total of 1500 genotypes. The phenotypic records consisted of PYT and AYT stages for last five years of the burn-in period, that were used for the genomic selection modelling to predict the breeding values. In the new subsequent year of the advancement phase, new genotypes were added from the PYT stage and phenotypic records from PYT and AYT generations are added to the training population. A ridge regression best linear unbiased prediction model “RR-BLUP” model (Hoerl and Kennard 1970; Meuwissen et al. 2001) was used for predicting the GEBVs. The prediction model was simulated, where genetic effects were obtained from marker effects and these effects were used to calculate the genetic variance. Residual error variance was modelled and weighed according to effective number of replications in each stage.

### Simulation of burn-in and the breeding advancement phase

#### Burn-in phase

**Fig. 3:**
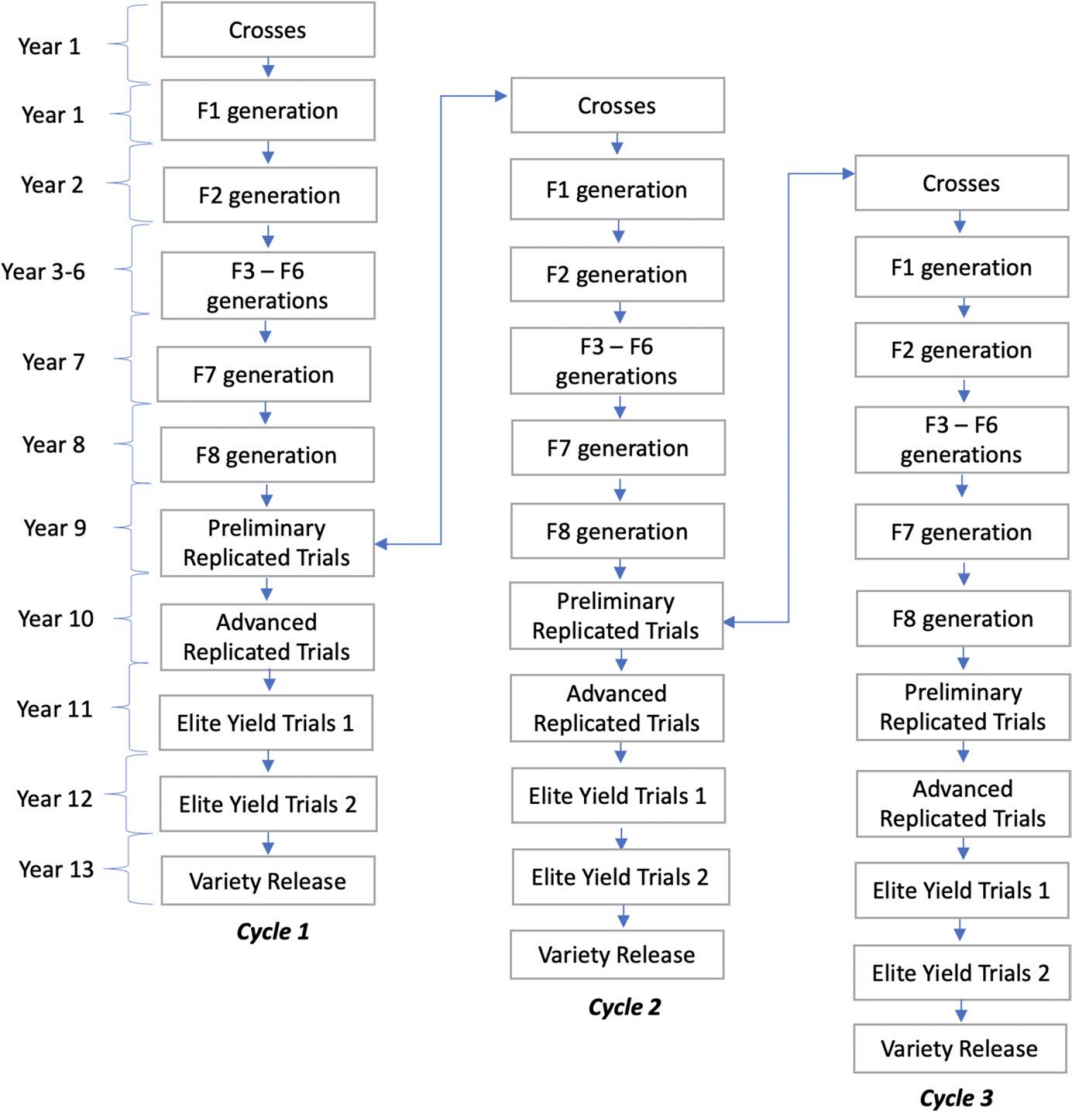
Schematic representation of updating the parents in the crossing block with the selected lines from the preliminary replicated trial stage of a previous cycle to the next, i.e. some of the old parents used in the previous year’s crossing block will be replaced with the best performed selected candidates from the PYT stage in the following year’s crossing block

The burn-in phase was simulated for a timespan of 20 years depicting a real conventional breeding program based solely on phenotypic selection. The design of the burn-in program was modelled as shown in the Fig. 1. The selection in the burn-in phase was performed using phenotypes. These phenotypes represented either direct selection for disease resistance or indirect selection for agronomic traits using visual selection on correlated traits (Fig. 3).

#### Breeding advancement phase

The breeding advancement phase was simulated for another 20 years following the burn-in phase using alternative breeding schemes. Each scheme was slightly modified based on the implemented selection method (phenotypic or genomic selection) and speed breeding (Fig. 2). Breeding cycle is represented in Fig. 3.

### Single trait and selection index-based selection approach, and comparison of breeding programs

#### Single trait and selection index-based selection approach

Two different approaches of selection were used in this study for selecting the individuals based on their trait performance: single trait approach and selection index approach with multiple correlated traits. In the single trait approach, DON content is used for selecting the individuals with the best performance of that generation. Whereas, in the selection index approach all the three correlated traits (FHB disease severity, DON content and AE) are used for selecting the individuals based on weights using Smith-Hazel index (Smith 1936). This index calculates the weights of traits based on their importance and purpose in breeding for new improved cultivars, considering the phenotypic and genotypic variance–covariance matrices. In this the genetic information from different traits can help selecting the candidates without ignoring other important traits. It is possible to increase and decrease the value of a particular trait in this index. The Smith-Hazel index is calculated as follows:

$$\text{b }= {\text{P}}^{-1}\text{Aw}$$

where P and A are phenotypic and additive genetic covariance matrices, respectively, and b and w are vectors of index coefficients and economic weighs, respectively. For each year the values of P and A are calculated using the above index of the respective population in that generation. Economic weights were (w = −1, 0, 0); i.e. major economic weight was given to the trait DON with a value of −1 and other traits were used as indicator traits using the trait correlations with DON. In this selection index approach, the selection candidate plants are selected using the given economic weights and correlations between the traits.

#### Comparison of breeding schemes

The potential and effectiveness of each breeding scheme can be determined based on comparisons among them used in the advancement phase. In general, the effectiveness of each breeding scheme was measured by comparing the genetic values of F8 generations over time in each of the corresponding schemes set to run for 30 iterations. Genetic values for this generation were examined because it is the important stage in which selection takes place to evaluate inbred lines after attaining homozygosity. In addition to the genetic values, we also monitored genetic gain, genetic variance, and accuracy of genomic predictions over time for the same stage of the schemes. Change of genetic gain and genetic variance in each of the breeding schemes was assessed by plotting mean genetic values of genotypes and variance of mean genetic values of F8 generation over time. To aid in visualization, the mean genetic values were centred at the mean value for F8 generation in Year 0 for each replicate. Year 0 was defined as the last year of the burn-in phase. Direct comparisons between breeding schemes for genetic gain and genetic variance were reported as ratios with 95% confidence intervals (95% CI). These were calculated by performing paired Welch’s t-tests on log-transformed values from the 30 iterations. The log-transformed differences and 95% CI from the t test were then back-transformed to obtain ratios (Ramsey and Schafer 2012). The accuracy of genomic predictions at each time point was assessed in each breeding scheme. Accuracy was defined as the correlation between the true genetic values and genomic estimated values of F8 generation. Accuracy was also estimated in the breeding scheme which did not implement genomic selection, where the accuracy was defined as a correlation between true genetic values and estimated phenotypic values of F8 generation. This was done only to enable comparisons across the alternative breeding schemes. The genomic estimated values themselves were only used for selection if breeding program design specified doing so. All these measures were also done in other generations of the breeding program as well to see the changes over time across the alternative breeding schemes.

## Results

### Genetic gain

**Fig. 4:**
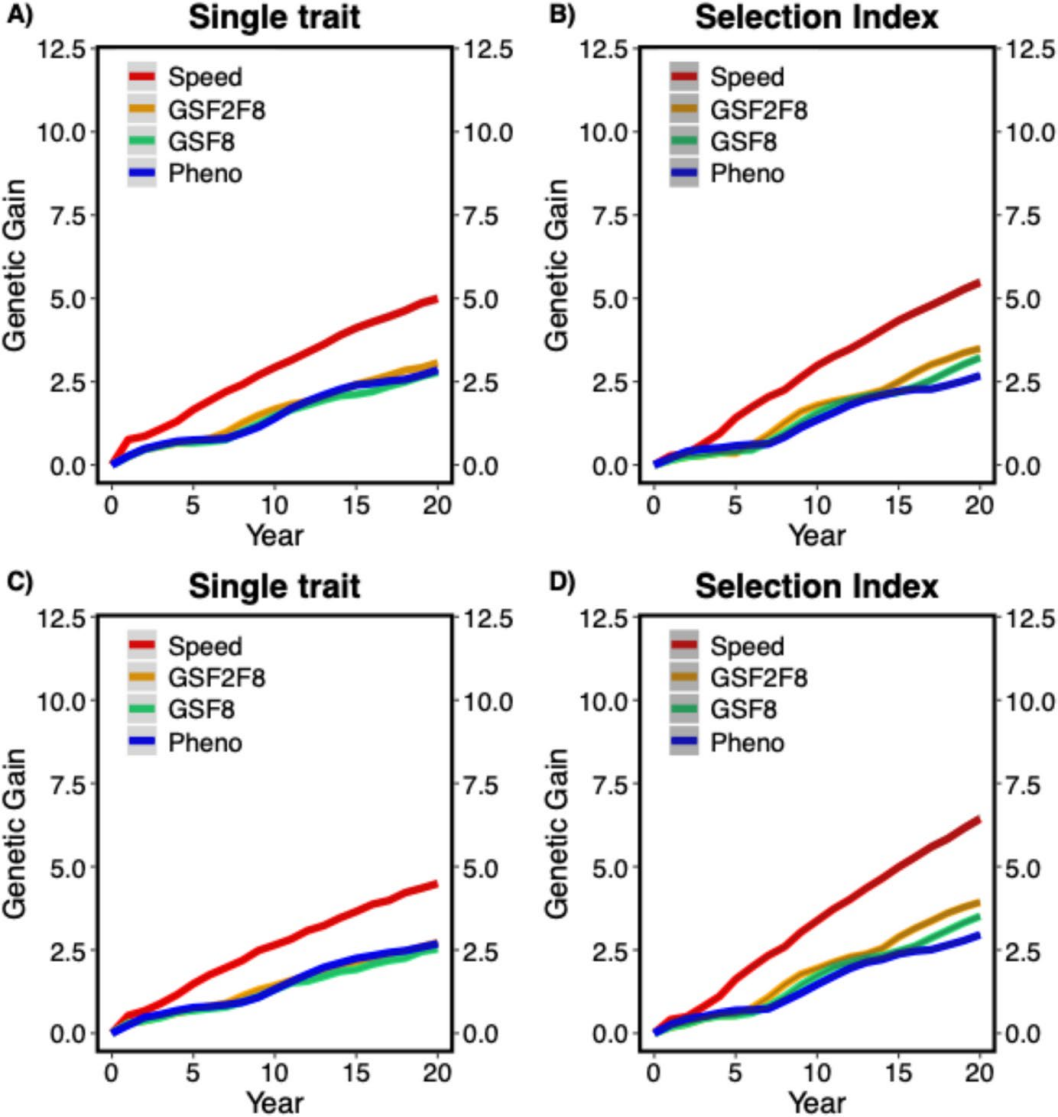
Genetic gain for three breeding schemes of breeding advancement phase; **A** MASBASIS – Single trait, **B** MASBASIS – Selection Index, **C** EXAMPLE – Single trait, and **D** EXAMPLE – Selection Index. Genetic gain is plotted as mean genetic value of F8 generation over time for 30 iterations. The lines within each box represents different breeding program scenarios and the shaded region indicates the standard error. Pheno; conventional phenotypic breeding scheme, GSF2F8; genomic selection F2-F8 breeding scheme, GSF8; genomic selection F8 breeding scheme and Speed; SpeedBreeding + GS selection breeding scheme

Genetic gain was measured as the mean genetic value of the trait DON content in the F8 generation over the entire period of breeding advancement phase. Genetic gain over time was higher for all the breeding program schemes with the selection index approach compared to the single trait approach. Genomic selection-based breeding schemes significantly increased the genetic gain compared to phenotypic selection. SpeedBreeding + GS and GSF2F8 produced faster and larger genetic gains for both MASBASIS and EXAMPLE datasets (Fig. 4).

Figure 4A and B show the change of genetic gain over time in the F8 generation for MASBABSIS tested with two approaches of selection (single trait and selection index). Genetic gain changes over time in the generation F8 for EXAMPLE dataset is shown in Figs. 4C and D (single trait and selection index). The selection index approach typically showed higher genetic gains compared to single trait selection in both datasets. For example, in MASBASIS dataset, a genetic gain ratio of 2.06 (95% CI [1.98, 2.14]) was produced when SpeedBreeding + GS compared with Pheno, that is higher than a ratio of 1.74 (95% CI [1.66, 1.83]) in single trait approach. Mostly, the selection index outperformed single trait across all the comparisons in MASBASIS and a similar pattern was observed with EXAMPLE dataset. Similarly, selection index approach records the highest genetic gain, with 2.18 (95% CI [2.12, 2.24]) for Speed vs Pheno in comparison to single trait ratio with 1.67 (95% CI [1.56, 1.78]). Additionally, genetic gain ratios in both the datasets are relatively close in values (Table 2).

**Table 2:** Comparisons between breeding programs for genetic gain and genetic variance as ratios with 95% confidence intervals (95% CI) in F8 generation for both MASBASIS and EXAMPLE datasets

	Genetic gain			Genetic variance		
	Speed vs Pheno	Speed vs GSF8	Speed vs GSF2F8	Speed vs Pheno	Speed vs GSF8	Speed vs GSF2F8
MASBASIS single trait	1.74 (95% CI [1.66, 1.83])	1.80 (95% CI [1.70, 1.90])	1.64 (95% CI [1.57, 1.71])	0.27 (95% CI [0.22, 0.34])	0.42 (95% CI [0.33, 0.52])	0.47 (95% CI [0.37, 0.60])
MASBASIS selection index	2.06 (95% CI [1.98, 2.14])	1.71 (95% CI [1.66, 1.76])	1.57 (95% CI [1.52, 1.62])	0.68 (95% CI [0.62, 0.74])	0.69 (95% CI [0.63, 0.74])	0.74 (95% CI [0.67, 0.81])
EXAMPLE single trait	1.67 (95% CI [1.56, 1.78])	1.78 (95% CI [1.65, 1.92])	1.66 (95% CI [1.54, 1.79])	0.27 (95% CI [0.21, 0.34])	0.41 (95% CI [0.29, 0.57])	0.48 (95% CI [0.37, 0.62])
EXAMPLE selection index	2.18 (95% CI [2.12, 2.24])	1.83 (95% CI [1.79, 1.88])	1.64 (95% CI [1.60, 1.67])	0.75 (95% CI [0.69, 0.81])	0.79 (95% CI [0.71, 0.88])	0.80 (95% CI [0.73, 0.88])

### Genetic variance

**Fig. 5:**
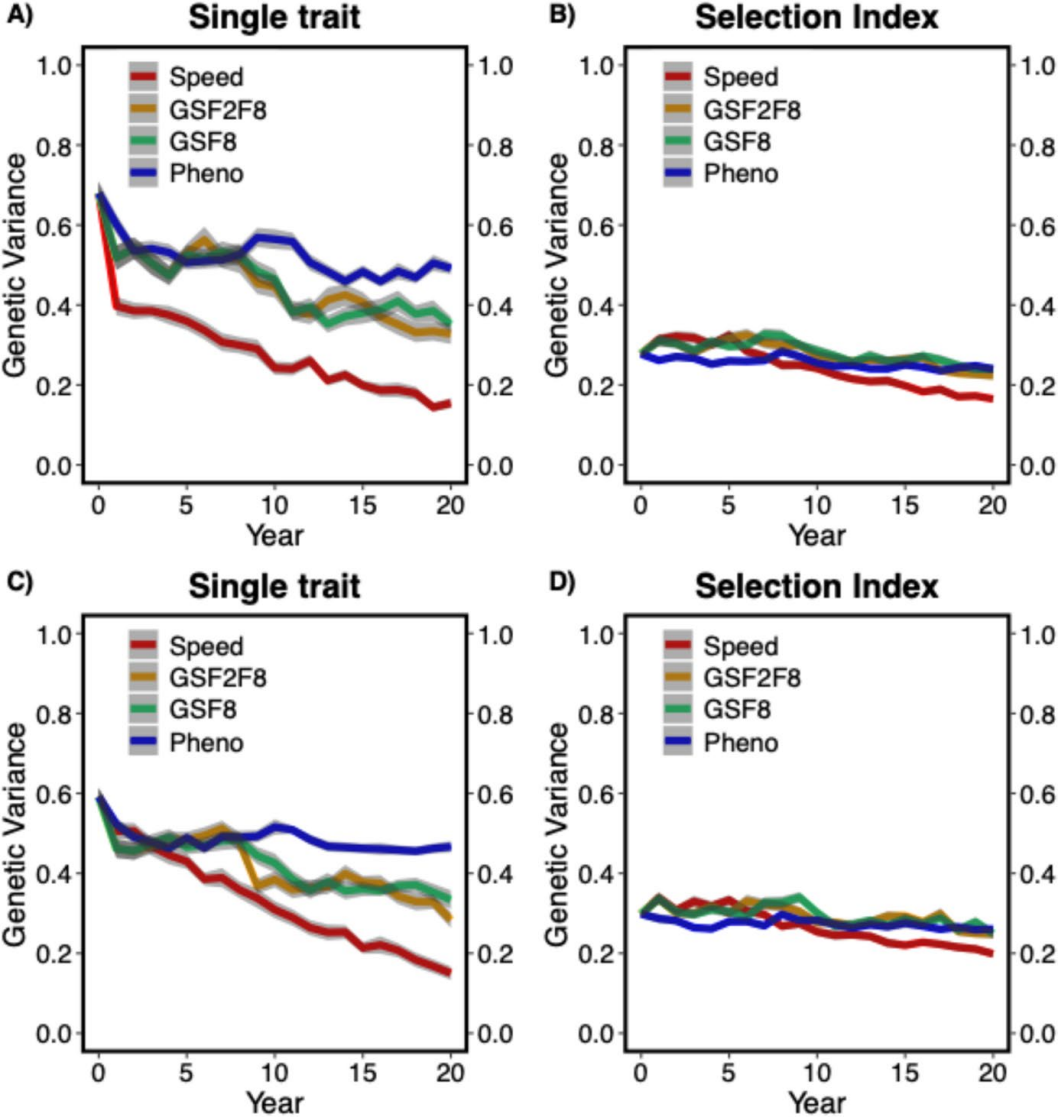
Genetic variance for four breeding schemes of breeding advancement phase; **A** MASBASIS – Single trait, and **B** MASBASIS – Selection Index, **C** EXAMPLE – Single trait, and **D** EXAMPLE – Selection Index. Genetic variance is plotted as mean genetic variance of F8 generation over time for 30 iterations. The lines within each box represents different breeding schemes and the shaded region indicates the standard error. Pheno; conventional phenotypic breeding scheme, GSF2F8; genomic selection F2-F8 breeding scheme, GSF8; genomic selection F8 breeding scheme and Speed; SpeedBreeding + GS breeding scheme

Genetic variance was measured as the mean genetic variance of the DON content in the F8 generation over the entire period of breeding advancement phase. Rapid decrease in the genetic variance was observed in the genomic selection based alternative breeding schemes compared to the phenotypic selection breeding scheme in case of the single trait approach. Whereas the reduction in the genetic variance in genomic selection breeding schemes was not very big compared to the phenotypic selection when the selection index approach was used. Mainly SpeedBreeding + GS showed a reduction in genetic variance compared to all other alternative breeding schemes in both single trait and selection index approaches for both MASBASIS and EXAMPLE datasets (Fig. 5).

In the Fig. 5A and B, change of genetic variance over time of all the breeding schemes in the breeding advancement phase is shown, tested for two approaches of selection (single trait and Selection Index) in MASBASIS dataset. The change of genetic variance over time in F8 generation for EXAMPLE dataset is shown in Fig. 5C and D, for single-trait and selection index approaches, respectively.

Selection index approach produced higher genetic variance ratios in comparison to single trait approach for MASBASIS and EXAMPLE datasets. In MASBASIS, selection index approach has a genetic variance ratio of 0.68 (95% CI [0.62, 0.74]) for SpeedBreeding + GS vs Pheno, whereas single approach showed a lower ratio of 0.27 (95% CI [0.22, 0.34]). Similarly, in EXAMPLE dataset selection index approach showed a higher genetic variance ratio with 0.75 (95% CI [0.69, 0.81]) for SpeedBreeding + GS vs Pheno, compared to 0.27 (95% CI [0.21, 0.34]) in single trait approach. Overall, selection index is advantageous over single trait approach across the dataset retaining higher genetic variance ratios (Table 2).

### Genomic prediction (selection) accuracy

**Fig. 6:**
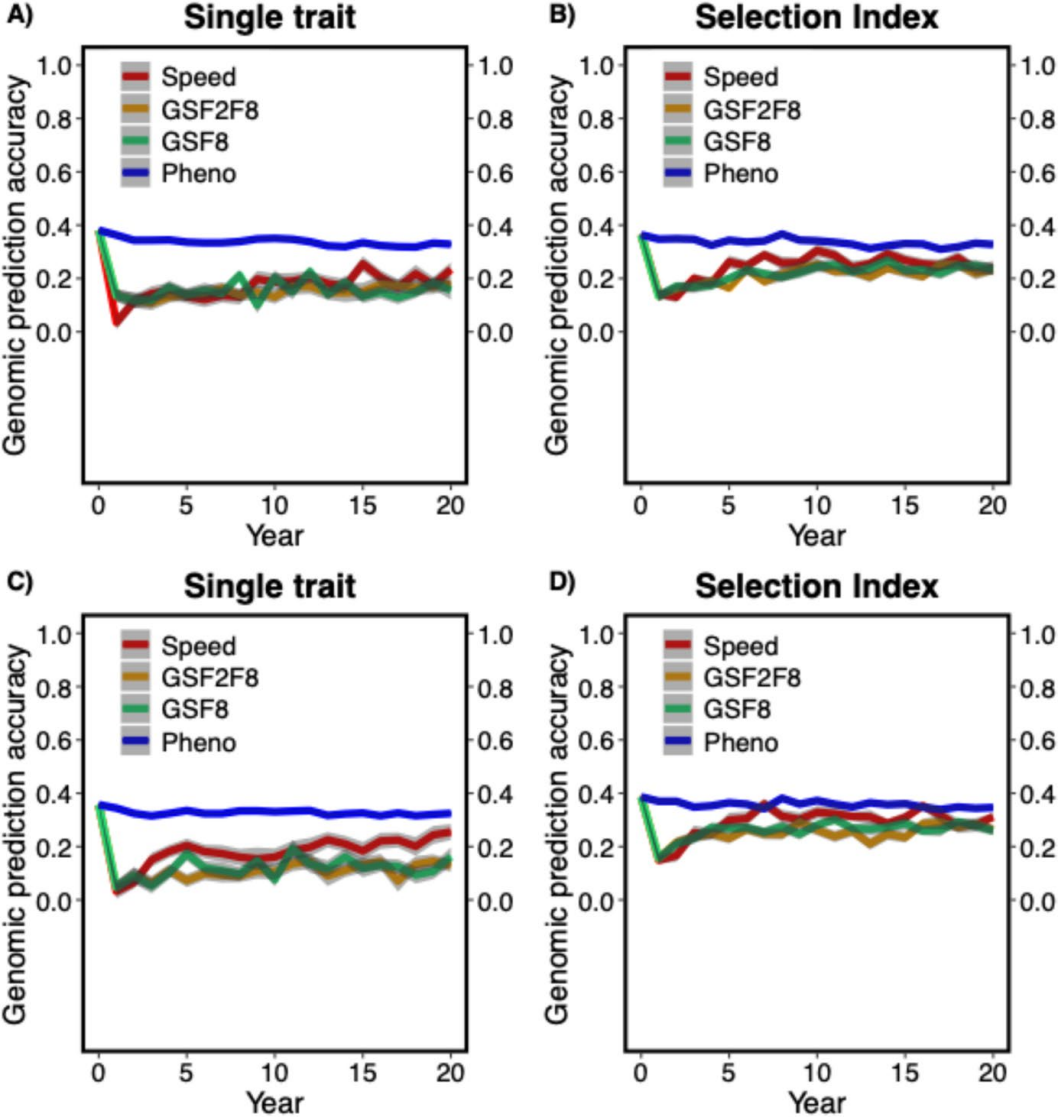
Genomic prediction (selection) accuracy for four breeding schemes of breeding advancement phase; **A** MASBASIS – Single trait, and **B** MASBASIS – Selection Index, **C** EXAMPLE – Single trait, and **D** EXAMPLE – Selection Index. Genomic prediction (selection) accuracy is the correlation between true and genomic predicted genetic values over time for 30 iterations. The lines within each box represents different breeding schemes and the shaded region indicates the standard error. Pheno; conventional phenotypic breeding scheme, GSF2F8; genomic selection F2-F8 breeding scheme, GSF8; genomic selection F8 breeding scheme and Speed; SpeedBreeding + GS breeding scheme

Genomic prediction (selection) accuracy was plotted as correlations between true and genomic predicted genetic values over time in the F8 generation over the entire period of breeding for the trait DON content. In Fig. 6, genomic prediction (selection) accuracy of all the breeding schemes over the breeding advancement is shown for the MASBASIS dataset. Figure 6A and B show the trends of genomic prediction accuracy change for single trait and selection index, respectively. Prediction accuracies for genomic selection increased over time in both the selection approaches but the only observed difference was that prediction accuracies were comparatively higher in the selection index approach (Fig. 6). Due to the information from individual phenotypes, phenotypic selection has the highest selection accuracy.

A similar result was observed in the EXAMPLE dataset for the change in prediction accuracies over time in F8 generation (Fig. 6C and D).

## Discussion

Increased genetic gain and faster development of new resistant cultivars or varieties requires breeding programs with reduced number of years per cycle or higher genetic gains per breeding cycle. This is possible with implementing genomic selection (Bančič et al. 2021; Faux et al. 2016; Gaynor et al. 2017; Voss-Fels et al. 2019a, b) and in addition speed breeding (Jighly et al. 2019; Voss-Fels et al. 2019a, b). Genomic selection and speed breeding implementation in plant breeding programs offers increased gains, but also brings additional costs to implement them. However, the genotyping costs are more affordable with reduced prices and alternative low-cost speed breeding tools are also available (Ghosh et al. 2018). To test the efficiency of the above mentioned methodologies in the breeding program, stochastic simulations are often used (Bančič et al. 2021; Gaynor et al. 2017; Kai Peter Voss-Fels et al. 2019a, b). In the same way, we tested the effectiveness of genomic selection and a combination of genomic selection and speed breeding to improve the FHB disease resistance in wheat breeding. We noticed from our simulations that implementing genomic selection in the breeding program contributed to a significant rise in genetic gain, but the genetic variance was significantly reduced over time. Advantages and disadvantages of the alternative breeding programs will be discussed here. In the first part of discussion, the impact of using genomic selection on change of genetic gain over time, change of genetic variance over time and change of selection accuracy (genomic prediction accuracy) over time. In addition, the impact of using genomic selection combined with speed breeding will be discussed with observed results and the role played by the selection strategies used in our study. Finally, the assumptions and limitations of the simulations, and whether these simulations are practical in real world breeding programs.

The discussion is based on four important aspects of the current study: i) Effect of implementing genomic selection on genetic gain, genetic variance and prediction accuracy, ii) Effect of combining speed breeding and genomic selection in the breeding scheme, iii) single trait vs selection index and iv) Assumptions and limitations.

### Effect of implementing genomic selection on genetic gain, genetic variance and prediction accuracy

Breeding schemes with genomic selection did generate higher genetic gain in all cases compared to the conventional phenotypic selection. Similar results were also reported in several other studies using stochastic simulations to observe the potential of genomic selection in their respective breeding programs (Gaynor et al. 2017, Voss-Fels et al. 2019a, b, Bančič et al. 2021). Gaynor et al. (2017) used stochastic simulations to test genomic selection in breeding programs for developing inbred lines using double-haploid technologies. They also showed how the two-part strategy for using genomic selection contributes to higher genetic gain than using standard genomic selection. Similarly, Bančič et al. (2021) evaluated the importance of genomic selection in intercrop breeding, they demonstrated similar results related to genetic gain.

The genetic gain showed a substantial increase across different genetic correlations used in the breeding program with genomic selection. In another study, Kai Peter Voss-Fels et al. (2019a, b) used simulations to evaluate the potential of an alternative breeding method called “SpeedGS” (a combination of speed breeding and genomic selection) in accelerating genetic gain. They showed that the genetic gain was much higher when speed breeding was combined with genomic selection compared to using genomic selection alone. The results seen in our study are in accordance with the results from these simulation studies in terms of increased genetic gain using genomic selection.

Shortening of breeding cycles is a key driver for increased genetic gain in all the alternative breeding schemes. The cycle interval of phenotypic selection is usually very long with intensive testing for many years. Although, the selection accuracies are high the selection cycle takes many years, which results in reduced genetic gains when compared to genomic selection. Genomic selection reduces the breeding cycle length in time, which results in higher genetic gain. This increased genetic gain in the breeding schemes using genomic selection is a result of shortened breeding cycle length and high prediction accuracies by using the RR-BLUP model (Meuwissen et al. 2001).

Breeders are not always confident about using genomic selection in breeding programs since the genotyping costs are high. But, in the recent years the genotyping costs have been reduced to an affordable level. Another constraint is lower prediction accuracies, but continuous efforts are being made to improve the prediction accuracies (Jarquín et al. 2017, 2014; Lopez-Cruz et al. 2015; Montesinos-López et al. 2018, 2021; Norman et al. 2017).

Genetic variance was decreased faster for all breeding schemes using genomic selection when compared to phenotypic selection. This reduction in the genetic variance in case of genomic selection breeding schemes is because of the high selection accuracy and reduced length of breeding cycles, resulting in an increased number of breeding cycles. This reduced genetic variance may be compensated by higher genetic gains, but this will not be beneficial when considering the long-term effects on the breeding program and germplasm. This long term disadvantage can be mitigated by adding genetic diversity to the breeding germplasm in the form of pre-breeding as described in the simulation study by Kai P. Voss-Fels et al. (2019a, b). Breeding schemes GSF2F8 and SpeedBreeding + GS showed similar reductions of genetic variance, because the stages in which genomic selection was used were the same in the generations of F2 and F8 (see Fig. 5). The change of genetic variance over time was also influenced by the selection method, which is discussed below.

Genomic predictions over time varied across different breeding schemes and when different selection strategies were used. The prediction accuracies followed an irregular pattern across the breeding schemes and they could be higher than in real-life breeding programs because we only considered additive effects; without dominance and epistasis into genomic prediction models (Forneris et al. 2017; Jiang and Reif 2015) and genotype-by-environment interactions were not taken into account as breeders normally do in their breeding programs (Gaynor et al. 2017; Jarquín et al. 2017; Mulder 2016). Other factors that affect the prediction accuracies in genomic selection are effective population size, training population size, relatedness, genetic distance between the training and prediction populations, and genetic architecture of traits and their correlations (Daetwyler et al. 2013, 2008; Hayes et al. 2009; Hickey et al. 2014). In our study, training population size increased every year in the breeding cycle and the number of phenotypic records, these factors would have caused the differences observed among the different breeding schemes. GSF2F8 and SpeedBreeding + GS breeding schemes showed similar genomic prediction accuracies, because the stages in which the genomic selection was conducted was the same in the generations of F2 and F8, this can be seen in Fig. 6.

### Effect of combining speed breeding and genomic selection in the breeding scheme

Combining genomic selection with speed breeding resulted in higher genetic gain compared to other genomic selection-based breeding schemes in our simulations. Combining speed breeding and genomic selection was also evaluated in a simulation study for increased genetic gains using a CIMMYT based wheat breeding program by Voss-Fels et al. (2019a, b). They concluded similar results in genetic gain as observed in our study, where the “SpeedGS” breeding scheme gave the highest genetic gain and outperformed other genomic selection-based breeding schemes. This idea of combining speed breeding with genomic selection is to further reduce the length of breeding cycle, that results in increased genetic gains based on the breeder’s equation, where gains are inversely related to generation time (Falconer and Mackay 1996). Speed breeding can achieve up to six generations of spring wheat per year under controlled greenhouse conditions (Watson et al. 2018). We used speed breeding to shorten the time to homozygosity, reducing the breeding cycle length to 7 years instead of 12, which was required for phenotypic selection. We followed the approach by which four generations of SSD could be completed in one year by using speed breeding (Voss-Fels et al. 2019a, b; Watson et al. 2018). Speed breeding is an expensive method which brings higher initial costs to the breeding program but is advantageous due to its faster turn-over of generations. However, there are cheaper protocols tested and published (Ghosh et al. 2018). These alternative protocols can be used in the breeding programs to achieve higher genetic gains by compromising considerable higher initial costs incurred to the breeding programs.

### Single trait vs selection index

We used two different strategies for selection of individuals based on the genetic architecture of fusarium head blight disease resistance and correlated traits (Bai et al. 2001; Buerstmayr and Buerstmayr 2015; Emrich et al. 2008; Hofgaard et al. 2016; Kubo et al. 2013; Lu et al. 2013; Skinnes et al. 2010, 2008; Snijders 2004). Real-life correlations observed in our previous GWAS results (Nannuru et al. 2022) were used in the simulations for selection, which is based on a selection index of multiple correlated traits. We used the Smith-Hazel index by giving all economic weight to the most important trait: DON. Genetic gains were higher when a selection index was used than in single trait selection. In the selection index approach, it is highly likely that correlations among the traits and the information borrowed from these multiple traits adds to the additional genetic gains observed in our results. Recently, a similar study by Dreisigacker et al. (2023) mentioned the agronomic traits measured but not selected for, are associated with the genetic gain for grain yield in wheat. This study recommended implementing genomic selection using multi-trait prediction models.

### EXAMPLE vs MASBASIS datasets

The results from two different simulated datasets were quite similar. But there is some observed difference in terms of ratios as shown in Table 2 and Supplementary table S1. We assumed the number of QTL or SNPs contributing to genetic architecture of the trait for MASBASIS dataset based on our previous GWAS study results (Nannuru et al. 2022) using the real-life genotypic data from 25 K SNP chip from TraitGenetics. For the EXAMPLE dataset, we assumed that all the available SNPs contributed to the genetic architecture of the trait. The results showed similar trends for changes of genetic gain over time, where overall genetic gain change for MASBASIS data was a little higher but we cannot conclude there is a significant difference between the two datasets in general. There is no apparent difference observed between the two datasets in cases of genetic variance changes over time and genomic prediction accuracies.

### Assumptions and limitations

Simulations conducted in our study did not model all the factors that are considered and included in a complex plant breeding program by the plant breeders. However, many important factors that are vital in the real-life breeding programs were considered and added. In the following part of the discussion, we describe the assumptions used for the simulation and their limitations. This includes the assumptions used for making the crosses, initial selection of morphological traits such as plant height and heading date and complexity of breeding goals. We assumed four generations of SSD per year based on the speed breeding method, which is less than the maximum of six generations possible for spring wheat as described by Watson et al. (2018). The assumptions will be discussed in detail below.

We assumed that the parents used in the simulation had no major differences in flowering time between crossing parents and that all crosses produced the required number of seeds. Whereas, in real-life practical breeding programs, differences in earliness between the parents can have an impact on the possible number of crosses.

Moreover, we assumed no major impact of earliness and plant height on FHB disease resistance, which is not true under a realistic breeding scheme since shorter plants which are desired agronomically tend to be more susceptible than tall ones and weather conditions during flowering (mainly warm and humid conditions) have high impact on obtained disease severity and DON content in disease nurseries. By using disease data adjusted for earliness and plant height (see materials and methods) as starting point for our simulations ensuring more accurate and reliable estimates of disease scores and toxin levels. Therefore, we do not expect that these potential confounding effects should have any major impact on the validity of our results.

In our simulation we mainly focused on the FHB disease related traits, whereas in the real breeding programs breeders have a wide variety of traits to be evaluated including major ones such as yield, agronomic and quality traits. However, we assumed the plants were selected for agronomically optimal plant height and heading date. In practice, these two traits can have big confounding effects on Fusarium infection levels, with tall plants being less affected by the disease and heading dates affecting disease levels depending of the weather conditions at anthesis with warm and humid conditions promoting Fusarium infections (Buerstmayr et al. 2020). By using disease data adjusted for plant height and days to heading to train the genomic prediction models we sought to cancel out these confounding effects by removing the confounding effects from the training data. An alternative approach would have been to model the two traits as correlated traits in multi-trait models.

Genomic selection has been used for various traits in wheat like yield, disease, and agronomic traits (Arruda et al. 2016; Jiang et al. 2017; Michel et al. 2017; Norman et al. 2017; J. A. Poland et al. 2012a, b; Rutkoski et al. 2014a, b; Song et al. 2017). For the real breeding programs most of these traits are needed to be evaluated together, and to do this a selection index is necessary. These selection indices with relevant economic importance need to be developed for a better multi-trait evaluation in the breeding program. In our simulation, we used the “Smith-Hazel index” (Smith 1936) with a high economic weight given to the trait DON content. Using this selection index, an increase in the genetic gain was obtained when compared to single trait selection, aided by the correlations with FHB and AE. Furthermore, there is a need to develop and define selection indices including all important traits in wheat breeding.

When combining speed breeding with genomic selection, the genetic gain could reach a plateau as it was observed in the simulation results by Kai P. Voss-Fels et al. (2019a, b) with a greater number of generations per year. To overcome this problem, pre-breeding can be a good alternative to keep the germplasm diversified.

## Conclusion

Using stochastic simulation, we found that genomic selection yielded higher genetic gains for FHB than phenotypic selection, which confirms the findings of other simulation studies. Combining genomic selection with speed breeding further enhanced the genetic gain. Hence, there is potential benefit to implement breeding schemes evaluated in our simulations in real-life breeding programs with the current availability of cheaper genotyping platforms and speed breeding protocols. Our simulations also showed that correlated multi-trait selection is advantageous over single trait with pre-defined weights based on the economic importance of the traits. Altogether, our results suggest that implementation of genomic selection in wheat breeding programs will be beneficial and give good returns for the investments despite the initial costs of implementing the technology. Future work should focus on implementing more factors in the simulations, such as Genotype x Environment interaction, population improvement, and other aspects from real-world breeding programs.

## Supplementary Information

Below is the link to the electronic supplementary material.

Supplementary file1 (DOCX 50 KB)

## Data Availability

The scripts and software used for data analyses in this study are available upon request.

## Abbreviations

AYT: Advanced Yield Trial
AE: Anther Extrusion
DON: Deoxynivalenol
EYT: Elite Yield Trial
FHB: Fusarium Head Blight
GEBVs: Genomic Estimated Breeding Values
PYT: Preliminary Yield Trial
GS: Genomic Selection
SB: Speed Breeding
SSD: Single Seed Descent
