# Supplementary material for: Evaluating genomic selection and speed breeding for Fusarium head blight resistance in wheat using stochastic simulations
Source: Mol Breed. 2025 Jan 9;45(1):14. doi: 10.1007/s11032-024-01527-z (PMC11717775; doi:10.1007/s11032-024-01527-z)
Supplement: Supplementary file 1 — Supplementary file1 (DOCX 50 KB) [file 11032_2024_1527_MOESM1_ESM.docx]

**Evaluating genomic selection and speed breeding for Fusarium head blight resistance in wheat using stochastic simulations**

Vinay Kumar Reddy Nannuru^1^, Jon Arne Dieseth^2^, Morten Lillemo^1^, Theo H.E. Meuwissen^3*^

^1^ Department of Plant Sciences, Norwegian University of Life Sciences, Ås, 1432 Norway

^2^ Graminor AS, Ridabu, 2322 Norway

^3^ Department of Animal and Aquacultural Sciences, Norwegian University of Life Sciences, Ås, 1432 Norway

# *Corresponding author: [theo.meuwissen@nmbu.no](mailto:theo.meuwissen@nmbu.no)

**Supplementary Figure 1** Genetic gain for four breeding schemes of breeding advancement phase; A) EXAMPLE – Single trait, and B) EXAMPLE – Selection Index. Genetic gain is plotted as mean genetic value of F8 generation over time for 30 iterations. The lines within each box represents different breeding program scenarios and the shaded region indicates the standard error. Pheno; conventional phenotypic breeding scheme, GSF2F8; genomic selection F2-F8 breeding scheme, GSF8; genomic selection F8 breeding scheme and Speed; SpeedBreeding+GS selection breeding scheme

**Supplementary Figure 2** Genetic variance for four breeding schemes of breeding advancement phase; A) EXAMPLE – Single trait, and B) EXAMPLE – Selection Index. Genetic variance is plotted as mean genetic variance of F8 generation over time for 30 iterations. The lines within each box represents different breeding program scenarios and the shaded region indicates the standard error. Pheno; conventional phenotypic breeding scheme, GSF2F8; genomic selection F2-F8 breeding scheme, GSF8; genomic selection F8 breeding scheme and Speed; SpeedBreeding+GS breeding program scheme

**Supplementary Figure 3** Genomic prediction (selection) accuracy for four breeding program schemes of breeding advancement phase; A) EXAMPLE – Single trait, and B) EXAMPLE – Selection Index. Genomic prediction (selection) accuracy is correlation between true and genomic predicted genetic values over time for 30 iterations. The lines within each box represents different breeding schemes and the shaded region indicates the standard error. Pheno; conventional phenotypic breeding program scheme, GSF2F8; genomic selection F2-F8 breeding scheme, GSF8; genomic selection F8 breeding program scheme and Speed; SpeedBreeding+GS breeding program scheme
